# A neuraminidase potency assay for quantitative assessment of neuraminidase in influenza vaccines

**DOI:** 10.1038/s41541-019-0099-3

**Published:** 2019-01-22

**Authors:** Rose T. Byrne-Nash, Jacob H. Gillis, David F. Miller, Katie M. Bueter, Laura R. Kuck, Kathy L. Rowlen

**Affiliations:** InDevR Inc., Boulder, CO, USA

## Abstract

Neuraminidase (NA) immunity leads to decreased viral shedding and reduced severity of influenza disease; however, NA content in influenza vaccines is currently not regulated, resulting in inconsistent quality and quantity of NA that can vary from manufacturer to manufacturer, from year to year, and from lot to lot. To address this problem, we have developed an assay for NA quantification that could be used by the industry to move toward developing influenza vaccines that induce a predictable immune response to NA. The VaxArray Influenza Seasonal NA Potency Assay (VXI-sNA) is a multiplexed sandwich immunoassay that relies on six subtype-specific monoclonal antibodies printed in microarray format and a suite of fluor-conjugated “label” antibodies. The performance of the assay as applied to a wide range of influenza vaccines is described herein. The assay demonstrated high NA subtype specificity and high sensitivity, with quantification limits ranging from 1 to 60 ng/mL and linear dynamic ranges of 24–500-fold. When compared to an enzymatic activity assay for samples exposed to thermal degradation conditions, the assay was able to track changes in protein stability over time and exhibited good correlation with enzyme activity. The assay also demonstrated excellent analytical precision with relative error ranging from 6 to 12% over day-to-day, user-to-user, and lot-to-lot variation. The high sensitivity and reproducibility of the assay enabled robust detection and quantification of NA in crude in-process samples and low-dose, adjuvanted vaccines with an accuracy of 100 ± 10%.

## Influenza vaccine: Multiplexed quantification of neuraminidase potency

Influenza vaccines that contain neuraminidase (NA) are associated with lower disease severity and better prognosis in vaccinated individuals, but the amount and quality of NA present in vaccines remains difficult to determine. Here, Rose Byrne-Nash and colleagues present the VaxArray Influenza Seasonal NA Potency Assay (VXI-sNA), a multiplexed sandwich immunoassay for the quantification of NA of all subtypes and for the determination of its potency. Featuring multiple NA subtype-specific antibodies printed in microarray format, the VXI-sNA showed high precision, dynamic range and reproducibility, and its results correlated well with NA enzymatic activity. This method is a step forward towards standardization of NA quantification for the assessment of stability, batch-to-batch variation and immunogenicity of NA in influenza vaccine formulations, and may help to develop influenza vaccines that trigger predictable immune responses to NA for increased protection against influenza infections.

## Introduction

There is increasing scientific evidence that neuraminidase (NA) within influenza vaccines leads to NA immunity, decreased viral shedding, and reduced severity of influenza disease.^1–12^ In a recent clinical trial, anti-NA immunity correlated more significantly with the reduction of all tested disease severity measures and had a stronger effect on prognosis than anti-hemagglutinin (HA) immunity.^11^ Trivalent influenza vaccines (TIVs) supplemented with purified NA were shown to be more effective than TIV alone in reducing pulmonary viral titers in mice following infection.^13^ Additionally, several recent studies have demonstrated that broadly reactive NA-directed antibodies can confer protection against a range of influenza subtypes.^3,14–17^ For example, it has been demonstrated that NA from seasonal H1N1 viruses may confer some protection against severe disease from exposure to avian H5N1 viruses during a pandemic.^15,18–20^ Given the overall disappointing efficacy of flu vaccines,^21–25^ such potential improvements in performance are highly desirable.

The amount of NA in influenza vaccines is largely unknown and unregulated, and thus current influenza vaccines are thought to contain NA of variable quality, quantity, and even lot-to-lot variability.^26^ At a World Health Organization (WHO) meeting in 2009 the lack of an assay and an appropriate NA standard were identified as the major hindrances to standardizing NA content in vaccines.^27^ More recently, the NAction! focus group consisting of NA-based immunity experts and industry leaders formed in order to promote NA research and a deeper understanding of how NA can contribute to the design of better, broadly protective vaccines. The group identified the need for a broadly available NA potency assay as a major hurdle that must be overcome for the standardization of NA content in vaccines.^26^

Enzymatic activity is typically used for verification of NA presence in influenza vaccines. Activity assays are problematic since different NA (sub)types can exhibit dramatically different enzyme kinetics and even small changes in buffer conditions can cause activity differences.^28^ Two alternative methods utilizing immunochemistry have been described recently.^29,30^ However, one of these methods was designed solely for the quantification of NA from H1N1 strains^29^ and another, while resistant to antigenic change due to the probing of a conserved, linear epitope, is not sensitive to changes in protein stability due to the requirement of degrading the NA protein before quantification.^30^

We previously reported that the VXI-sNA is predictive of NA immunity for an N2 subtype within an H3N2 monovalent vaccine.^31^ Herein, we summarize the development and overall performance of the multiplexed NA potency assay for all NA subtypes (N1, N2, and B-NA) in seasonal influenza vaccines produced by a wide range of manufacturing methods. The aim of this work is to address the critical and unmet need for a standardized NA quantification method capable of tracking changes in stability and immunogenicity. Very little monoclonal antibody (mAb) is required for each assay, allowing a typical antibody production run to produce enough antibody for the quantification of over 500,000 samples. This reagent sparing approach overcomes the issues around reagent scarcity outlined in the recent NAction! report^26^ and offers a potential solution for users across the influenza vaccine industry to standardize their method and reagents for NA quantification.

## Results

### Development of VXI-sNA assay

The first step in developing VXI-sNA was to screen a panel of mAbs and to select those with the desired specificity, sensitivity, and stability indication properties. Antibodies that demonstrated high coverage, high specificity, and a reduction in signal upon thermal degradation were down-selected for inclusion in the final version of the array (Supplementary Figure 1 and 2).

### Assay principles

**Fig. 1:**
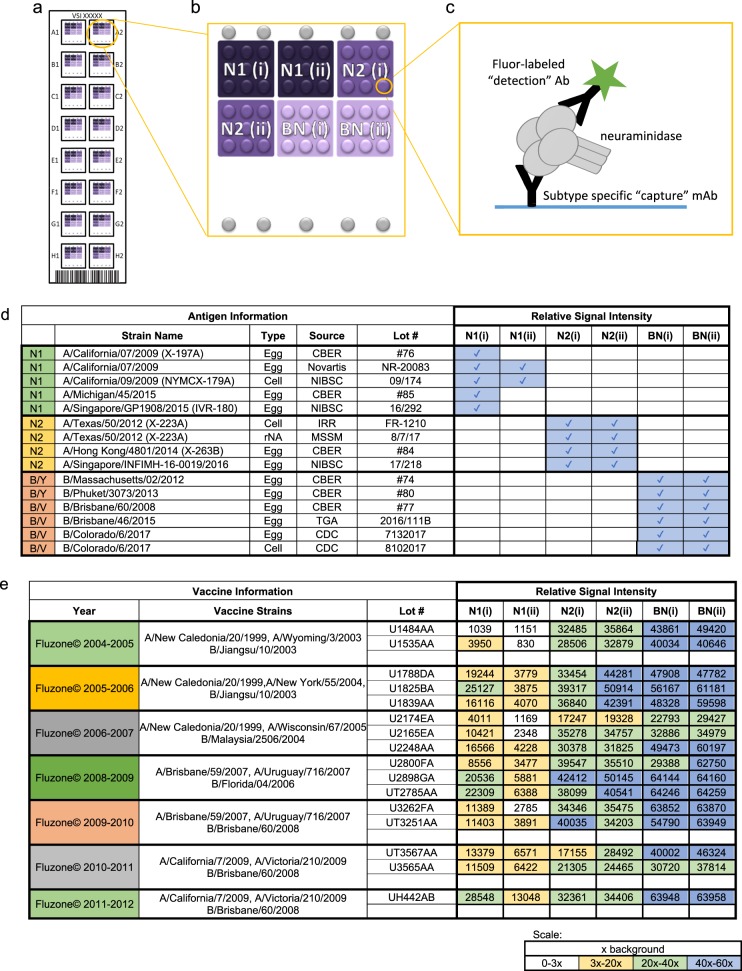
VaxArray Influenza Seasonal Neuraminidase Potency Assay (VXI-sNA). **a** Illustration of a VXI-sNA microarray slide containing 16 identical microarrays in 16 wells. **b** Schematic of the VXI-sNA array layout of subtype-specific antibodies to N1, N2 subtypes and B-NA. Two different monoclonal antibodies (mAbs) are printed for each subtype and distinguished from one another using (i) or (ii) labeling. The array contains nine replicate spots (~200 µm in diameter) of each monoclonal antibody. **c** Immunoassay schematic. **d** Qualitative assessment of VXI-sNA capture mAbs for detection of a panel of recent vaccine strains produced across multiple production platforms with a blue box and checkmark to indicate mAb detection of the listed antigen. Detection was defined as 3× the background intensity. **e** Signal intensities for each VXI-sNA capture mAb against a panel of historical FluZone vaccines from 2004 to 2012. White/empty boxes indicate signal intensity below the 3× background intensity cutoff, yellow indicates signal intensity between 3× and 20× background intensity, green indicates between 20×–40× background, and blue indicates between 40× and fluorescence saturation

VXI-sNA is a multiplexed sandwich immunoassay that consists of subtype-specific mAbs printed in a microarray format illustrated in Fig. 1a, b. There are two capture mAbs per subtype. For identification purposes, each capture mAbs is identified as (i) or (ii) as displayed in Fig. 1b. There are 16 such microarrays per slide. To run the assay, a monovalent or trivalent standard is prepared and a series of dilutions are analyzed on the left 8 arrays of the VXI-sNA slide (Fig. 1a) to generate a standard curve that the samples, run on the right 8 arrays, are calibrated against. On each array, NA protein is captured by the printed mAbs before a fluor-conjugated label antibody is added for detection (Fig. 1c). The spatial separation and high subtype specificity of the capture mAbs allows for the multiplexed analysis of multivalent vaccines. The assay is NA specific and not affected by the high concentrations of other viral proteins like HA (Supplementary Figure 3).

### VXI-sNA is responsive to the latest vaccine strains for all licensed production platforms

A panel of 15 antigens from the last five northern hemisphere flu seasons, produced via egg-derived, cell-derived, and recombinant manufacturing, was tested by VXI-sNA to evaluate the compatibility of the assay with the latest strains produced via all of the currently licensed production platforms. Because these samples did not have predetermined NA concentrations, each sample was diluted based on HA concentration and analyzed by VXI-sNA individually. Because a fixed amount of NA could not be assessed for each sample, only whether or not the sample was detected is reported (Fig. 1d). Detection was defined as 3× the background signal intensity. All samples were detected (15/15) by one or more VXI-sNA capture mAb. Additionally, no cross-reactivity was observeed for any of the capture mAbs. In other words, N1 antibodies only detected N1 antigens and so on. This study demonstrates that VXI-sNA is capable of detecting and potentially quantifying NA potency of the current 2018–2019 Northern hemisphere vaccine strains that were announced in February of 2018, A/Singapore/GP1908/2015 (H1N1), A/Singapore/INFIMH-16-0019/2016 (H3N2), B/Phuket/3073/2013 (B/Y), and B/Colorado/06/2017 (B/V). The VXI-sNA was not affected by recent strain changes, for example, the H3N2 strain changes from A/Texas to A/Hong Kong to A/Singapore that occurred over the past 4 years and the recent change from B/Brisbane to B/Colorado. This study also demonstrated the subtype specificity for each of the capture mAbs, with none of the VXI-sNA capture antibodies exhibiting signal above 3× background for non-target antigens. While the assay was able to detect NA from both the B/Yamagata and the B/Victoria lineages, the BN antibodies were not able to distinguish between NA from the two lineages, likely due to a reassortment event that occurred in the early 2000s resulting in the both lineages encoding B-Yamagata-like NA.^1,32^

### VXI-sNA is robust against antigenic drift

As with HA, antigenic drift occurs in NA.^33,34^ A successful potency test for NA potency needs to be somewhat resistant to evolutionary changes in the protein to avoid constant changes to the assay for each strain change. To evaluate the performance of the VXI-sNA assay with respect to strain changes, we tested a panel of vaccines that spanned 8 years and nine strain changes. As summarized in Fig. 1e, all NA components (N1, N2, and B-NA) were detected for the trivalent FluZone vaccines produced between 2004 and 2012, with the exception of the A/New Caledonia/20/1999 N1 component of the 2004–2005 vaccine. Since the N1(i) mAb detects NA from seven other vaccines that contain the A/New Caledonia/20/1999 strain, we speculate that the N1 component of this vaccine was low in that production lot or had degraded substantially. We were not able to test this speculation by running an activity assay because the 2004–2005 vaccine is a trivalent mixture and we would not be able to distinguish the activity of the A/New Caledonia antigen from the activity of the NA molecules from the other (sub)types within the mixture.

We noticed lower signals across N1 antigens for the N1(ii) antibody. This is likely because N1(ii) was raised against A/Puerto Rico/8/1934. This antibody was included in the array to quantify older vaccine strains and for applications not explored in this manuscript.

### NA standards for quantification of NA

Currently, there is no influenza-specific NA calibration standard available. Therefore, to perform a quantitative assessment of VXI-sNA, a standard must be defined. NA “standards” for enzymatic assays such as the 2′-(4-methylumbelliferyl)-α-d-*N*-acetylneuraminic acid (MUNANA)-based assays are typically from non-influenza species (e.g., *Clostridium perfringens*, *Vibrio cholerae*, and *Arthrobacter ureafaciens*) and are used only for relative activity (typically, units are defined by the amount of enzyme that will catalyze the release of 1µmol sialic acid per min under assay conditions). While a recombinant NA standard seems like a simple solution to the lack of an NA standard, we and several others have demonstrated that recombinant proteins (especially in the case of glycosylated proteins, such as HA and NA) interact with mAbs with different avidity than native proteins.^35^

As one possible approach for NA standardization, we tested the utility of the current reference reagents for HA, which are whole inactivated viruses, to also serve as reference reagents for NA. The assumption is that all of the NA content is in an “active” form within the whole virus, such that an absolute method measuring total protein could be used to determine NA content. One absolute method that has been established for both HA and NA is isotopic dilution mass spectrometry (IDMS).^7,36,37^ Therefore, the NA content was determined by IDMS through a collaboration with the US Centers for Disease Control and Prevention (CDC) for the following *whole virus* CBER Reference Reagents from the 2016–2017 flu season: A/California/07/2009 (Lot # 76), A/Hong Kong/4801/2014 (Lot # 84), B/Brisbane/60/2008 (Lot # 77), and B/Phuket/3073/2013 (Lot # 80), which were then used as standards in all quantitative studies described in this work.

### The linear dynamic range of VXI-sNA is more than 120-fold

**Fig. 2:**
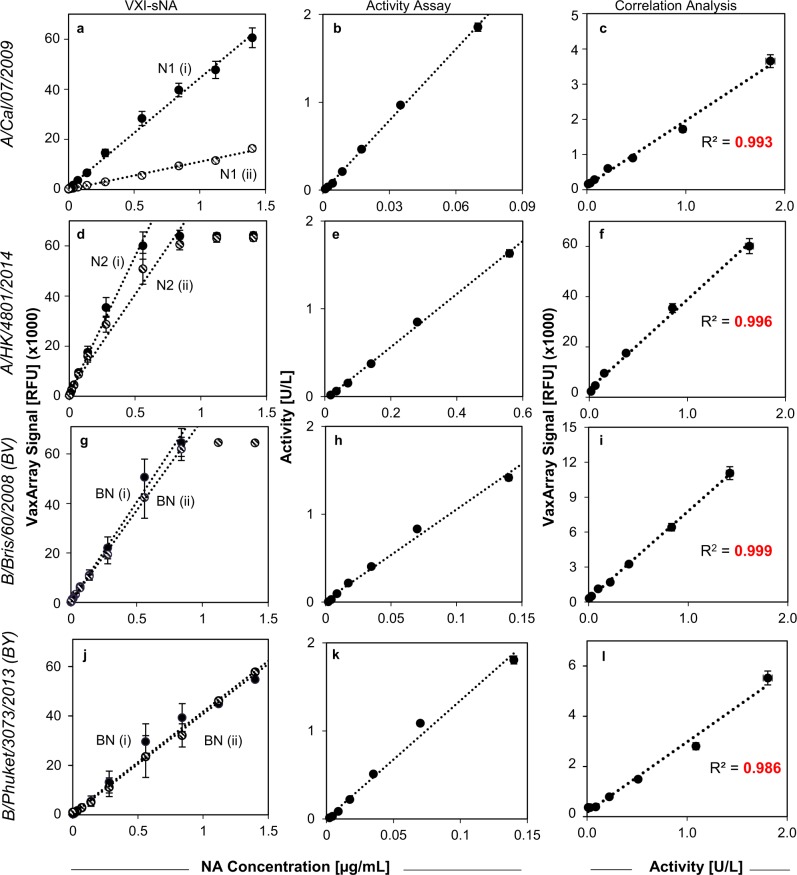
Linear dynamic range comparison of VaxArray Influenza Seasonal NA Potency Assay (VXI-sNA) and a neuraminidase (NA) activity assay. Serial dilutions of four antigens were analyzed by VXI-sNA and an NA activity assay. VXI-sNA response curves, enzymatic activity response curves, and a correlation plot of one of the VXI-sNA capture antibody vs. NA activity are shown, respectively, for H1N1 A/California (CBER, Lot # 76) (**a**–**c**), H3N2 A/Hong Kong (CBER, Lot # 84) (**d**–**f**), B/Brisbane (CBER, Lot # 77) (**g**–**i**), and B/Phuket/3073/2013 (CBER, Lot # 90) (**j**–**l**). For correlation plots (**c**, **f**, **i**, **l**), the signal responses for N1(i), N2(i), and NB(i) are plotted against the corresponding activity response. Error bars for VXI-sNA represent the standard deviation of the nine antibody spots for the corresponding capture antibody for each array. Error bars for the enzymatic activity represent previously determined representative error of the assay (2.5%)

The CBER reference antigens with NA concentrations determined by IDMS were tested by VXI-sNA over a range of 0.001–1.40 µg/mL in 13-point dilution series to evaluate the capture mAbs for linear dynamic range (LDR) as well as upper and lower limits of quantification. The resulting response curves are presented in Fig. 2a, d, g, j. All VXI-sNA capture antibodies produced robust, linear responses to increasing antigen concentrations. Using each VaxArray response curve, lower quantification limits, upper quantification limits, and LDRs were calculated and reported in Table 1. The nanogram-level lower limits of quantification allow the user to run dilute samples, an advantage for an assay that quantifies NA, which is a minor component in influenza vaccines compared to HA. This also allows for substantial dilution of samples, providing the ability to reduce the concentration of potentially interfering compounds found in in-process samples and adjuvanted vaccines.

**Table 1:** Quantification ranges for VXI-sNA and activity assay

Subtype	mAb ID	Lower QL (µg/mL)	Upper QL (µg/mL)	Range
N1	N1(i)	0.011	>1.4	>129×
	N1(ii)	0.059	>1.4	>24×
	Activity	0.001	0.07	54×
N2	N2(i)	0.003	0.56	215×
	N2(ii)	0.006	0.84	145×
	Activity	0.017	0.56	33×
B/V	BN(i)	0.001	0.84	570×
	BN(ii)	0.003	0.84	305×
	Activity	0.002	0.14	77×
B/Y	BN(i)	0.03	>1.4	>46×
	BN(ii)	0.03	>1.4	>46×
	Activity	0.002	0.14	77×

*mAb* monoclonal antibody

Differences were observed in the avidity of the BN mAbs for NA from B/Victoria-like and B-Yamagata-like lineages. This suggests that the NA from the two lineages, while similar enough to be detected by the same mAbs, have some degree of difference from one another in the region of the NA protein that the BN mAbs probe.

### VXI-sNA correlates with activity but has a larger LDR

Typically, NA enzymatic activity assays determine the catalytic activity of NA by measuring the accumulation of a fluorescent product cleaved from a non-fluorescent substrate by NA. While the NA activity assay has its shortcomings (strongly affected by buffer conditions, non-homogenous standards, etc.), it has been shown that NA activity correlates well with a protective immune response^7,31^, and as such, it is important that any potency assay demonstrate correlation with enzymatic activity. To compare VXI-sNA to the commonly used MUNANA or MUNANA-like enzymatic assays, the same 13-point reference antigen dilution series preparations analyzed by VXI-sNA to determine LDR were also simultaneously analyzed by a MUNANA-like activity assay (Sigma, Cat # MAK121). The MUNANA-like activity assay produced a linear response to increasing NA concentrations up to 0.070, 0.56, and 0.14 µg/mL for N1, N2, and NB, respectively (Fig. 2b, e, h, k). Reference antigen preparations above these concentrations produced fluorescent signal higher than the internal, non-influenza NA standard used to calibrate the activity assay or were at fluorescent saturation and thus are not presented. When compared side-by-side to VXI-sNA, both assay demonstrated comparable nanogram-level lower limits of quantification; however, VXI-sNA demonstrated higher upper limits of quantification than the activity assay, resulting in much broader LDRs (Table 1).

For concentrations of each sample tested that fell within the LDR of both assays, the VXI-sNA signal was plotted against the corresponding MUNANA measured activity and the resulting correlation plots are presented in Fig. 2c, f, i, l. An obvious correlation between VXI-sNA and enzymatic activity was observed, with Pearson's correlation coefficients (*R*^2^) between activity and VXI-sNA signal of 0.993 and 0.955 for N1(i) and N1(ii), 0.996 and 0.996 for N2(i) and N2(ii), and 0.999 and 0.997 for BN(i) and BN(ii), respectively. The high degree of correlation indicates that VXI-sNA is probing enzymatically active, conformationally intact protein.

### VXI-sNA demonstrates a high level of precision

**Fig. 3:**
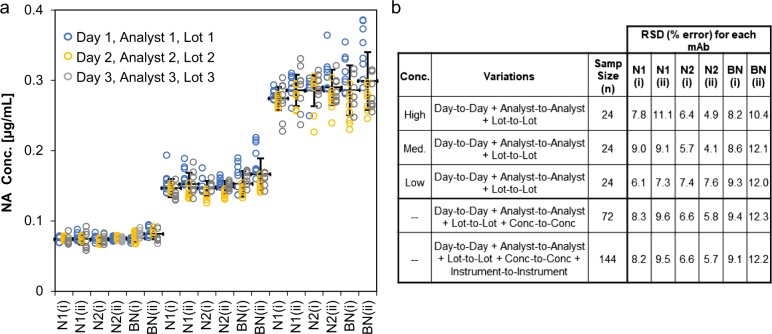
Precision. A trivalent mixture of H1N1 A/California (CBER, Lot # 76), H3N2 A/Hong Kong (CBER, Lot # 84), and B/Phuket (CBER, Lot # 80) antigen was diluted to three concentrations and analyzed in eight replicates against a standard curve generated from the same trivalent mixture. All concentrations were normalized to the N1 concentration. The experiment was performed by three separate users, on three separate days, with three separate reagent lots, represented by the three different colored points in **a**. The average neuraminidase (NA) concentration of all 24 replicates across all 3 days for each antibody is shown as a thick black bar. Error bars represent the standard deviation of all 24 replicates. **b** The relative standard deviation (RSD) is shown for various comparisons, including the high-, medium-, and low-concentration samples, the overall day-to-day variability, and the overall variability including scanning the same slides using different VaxArray Imaging Systems

To assess the precision of VXI-sNA, a trivalent standard was measured by VXI-sNA at three different concentrations to represent the upper, middle, and lower end of the LDR of each mAb. Consistent with recommendations by the International Conference on Harmonization Guideline for Validation of Analytical Procedures,^38^ assay performance was tested by three different operators, on three different days, using three different production lots of VXI-sNA microarray slides, three different production lots of all other assay reagents, and two different VaxArray Imaging Systems. Because each capture mAb has a different LDR (see Fig. 2j), the concentrations selected for each mAb varied and thus were normalized to the concentration of the N1 antigen before being displayed in the univariate scatterplot in Fig. 3a. Relative standard deviations (RSDs) were calculated in a number of ways, including (i) for each mAb at each concentration, (ii) for each mAb with the three concentrations pooled, and (iii) for each mAb with the three concentrations on both instruments pooled (Fig. 3b). The average error of the six mAbs combined for each day were 7.5 ± 2% for day 1, 5.5 ± 2% for day 2, and 7.9 ± 3% for day 3, and thus there were no significant differences in observed error from day-to-day. The concentrations tested at the upper and lower limits of the LDR of each mAb did not differ significantly from the concentration at the middle of the LDR, highlighting the precision of the assay across a wide range of concentrations. The relative error for all of the capture mAbs ranged from 6 to 12%, taking into account all of the tested variations (i.e., worst-case scenario). While there is no gold standard to compare the precision of VXI-sNA to, the single radial immunodiffusion assay for HA quantification has a relative error of ~12%. Therefore, VXI-sNA has a level of precision that is similar or better than the current gold standard for HA quantification.

### VXI-sNA is capable of tracking protein stability

**Fig. 4:**
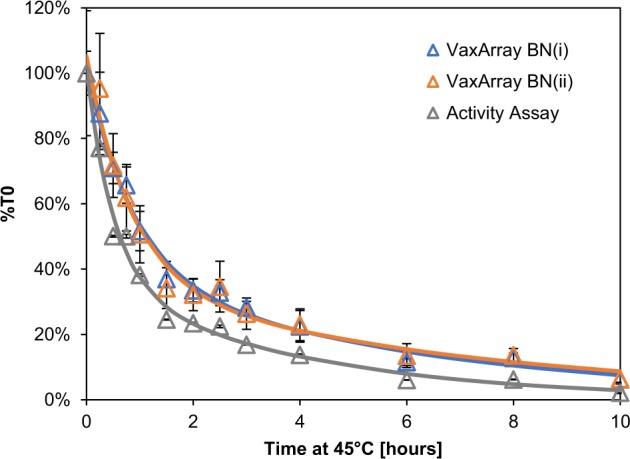
VaxArray Influenza Seasonal NA Potency Assay (VXI-sNA) and neuraminidase (NA) activity assay response to thermally degraded antigen. A B/Phuket sample (CBER, Lot # 80) was incubated at 45 °C for up to 10 h. After degradation, each time point was analyzed by VXI-sNA and an NA activity assay and the NA concentration and activity measured in replicate of three. The average %T0 (assay response at the time point divided by the T0, non-degraded, sample response) is plotted for each time point for the NB(i) monoclonal antibody (mAb) (blue curve), NB(ii) mAb (orange curve), and NA activity assay (gray curve). Error bars represent the standard deviation of the triplicate measurements of each time point

To provide further support that VXI-sNA is quantifying conformationally active NA protein, we performed a thermal forced degradation study using a B/Phuket/3073/2013 reference antigen (CBER, Lot # 80). The antigen was aliquoted and all aliquots were exposed to 45 °C. The behavior over a 10-h period was tracked by removing aliquots and testing them by VXI-sNA and an off-the-shelf NA activity assay. Both assays demonstrated a rapid decrease in measured response followed by a slower fall in signal/activity, with excellent correlation (Fig. 4). A two-phase decay model was fit to each dataset using a regression derived from a method described previously^28^ and the half-lives and decay rates compared. The decay rates as measured by signal on antibodies BN(i) and BN(ii) were the same (*k*_fast_ of 1.20 ± 0.28 and 1.21 ± 0.35, and *k*_slow_ of 0.17 ± 0.02 and 0.15 ± 0.02 for BN(i) and BN(ii), respectively). The activity assay exhibited a slightly faster rate of decay, with a *k*_fast_ of 1.94 ± 0.02 and a *k*_slow_ of 0.25 ± 0.002, potentially indicating somewhat higher sensitivity to protein structure. Because this study was only performed using a B/Phuket antigen, we can only conclude that the B-NA mAbs are capable of tracking changes in protein confirmation as related to enzymatic activity. However, this finding along with the stability study presented in Supplementary Figure 2 indicate that this capability is very likely conserved across all of the capture mAbs in the VXI-sNA assay, suggesting that the assay can be used to allow vaccine manufacturers to monitor the stability of NA in their vaccines and even help them formulate their vaccines for optimal NA stability.

### VXI-NA is capable of quantifying crude in-process samples

**Fig. 5:**
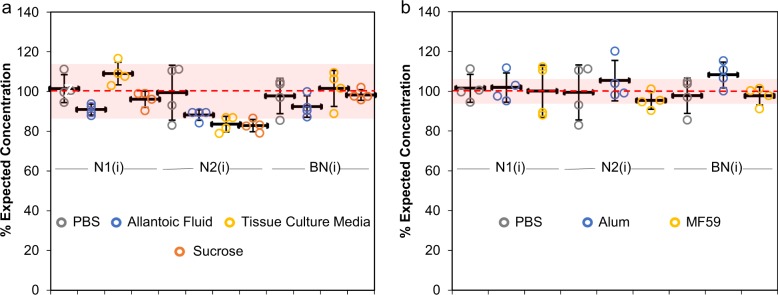
Quantification of neuraminidase (NA) by VaxArray Influenza Seasonal NA Potency Assay (VXI-sNA) in the presence of common interfering agents. The following antigens were spiked into phosphate-buffered saline (PBS), allantoic fluid, 40% sucrose, and exhausted Dulbecco’s modified Eagle’s medium (DMEM) + 10% fetal bovine serum (FBS) medium from uninfected Madin–Darby Canine Kidney (MDCK) cells (tissue culture media) to a final concentration of 2 µg/mL and then analyzed by VXI-sNA: H1N1 A/California (CBER, Lot # 76), H3N2 A/Hong Kong (CBER, Lot # 84), and B/Phuket (CBER, Lot # 80). The VXI-sNA measurements for each capture antibody were then divided by the expected concentration of 2 µg/mL and multiplied by 100 to generate “% Expected Concentrations” that were then plotted individually for each replicate **a**. VXI-sNA potency determination for influenza antigens spiked into common adjuvants such as aluminum hydroxide (alum) and MF59 is shown in **b**. For each sample, a PBS-negative control was included. For both **a** and **b** each data point represents a single replicate. The thick black bar represents the average across the four replicates. Error bars represent the standard deviation across the four replicates for each sample. The red-dotted line represents the 100% expected concentration for each sample. The shaded red region represents 100% expected concentration plus and minus 10% for **a** and 5% for **b**

To assess the ability of VXI-sNA to track antigen within upstream vaccine manufacturing steps, we spiked a trivalent mixture of reference standards (whole virus) into (i) uninfected allantoic fluid from 10-day-old embryonated chicken eggs to simulate egg-based vaccine samples at harvest, (ii) exhausted tissue culture media from Madin–Darby Canine Kidney (MDCK) cells to simulate cell-based vaccine samples at harvest, and (iii) 40% sucrose to simulate vaccine samples after sucrose gradient purification. Preparation and dilution of the reference standards to generate the “mock” in-process samples was based upon their HA content to best mimic real-world applications. To this end, each sample was spiked into each medium and into phosphate-buffered saline (PBS) (as a negative control) at a final concentration of 10 µg/mL HA and lysed at room temperature for 30 min before analysis by VXI-sNA. The resulting NA concentrations were compared to the expected NA concentrations, which were calculated from the known IDMS-determined stock NA concentrations and the performed dilution factors. Results are reported in Fig. 5a as % Expected Concentration for each capture mAb. All of the VXI-sNA individual capture mAbs exhibited good agreement with the expected concentration (±10%) in the presence of crude matrices, with the exception of the N2 samples that demonstrated slightly lower recovery for allantoic fluid (88 ± 3%), tissue culture media (83 ± 4%), and sucrose (82 ± 3%) (Fig. 5a). Despite the lower recovery, these samples were within error of the N2 PBS control (99 ± 14%), and thus could be low simply due to variation within the assay. Overall, these results support the assertion that VXI-NA could provide vaccine manufacturers with a simple way to monitor NA yield throughout the process, including seed strain optimization, propagation, harvest, and at various purification steps.

### VXI-sNA is capable of quantifying low-dose adjuvanted vaccines

To evaluate the ability of VXI-sNA assay to quantify NA content in dose-sparing, adjuvanted vaccines, we formulated mock vaccines containing low antigen levels in industry-relevant adjuvants and analyzed them by VXI-sNA. Each mock vaccine consisted of a final concentration of 5 µg/mL of HA and 1.7 mg/mL elemental aluminum (in alum) or 19.5 mg/mL squalene (from MF59). These concentrations were selected based on previously published, recommended adjuvant concentrations for alum and MF59.^39^ A non-adjuvanted negative control sample was included where PBS was used instead of adjuvant. These mock adjuvanted samples were held at room temperature for 30 min to mimic real-world bed-side preparation of an adjuvanted vaccine before being analyzed by VXI-sNA. An 8-point trivalent standard curve was prepared from antigen spiked into PBS. The resulting NA concentrations were compared to the expected NA concentrations (calculated from the known IDMS-determined stock NA concentrations and the performed dilution factors) and reported in Fig. 5b as % Expected Concentration for each capture mAb. All of the VXI-sNA capture mAbs exhibited good agreement with the expected concentration (±5%) (Fig. 5b, red box) despite the presence of alum-based or squalene-based adjuvant. These results support an assertion that VXI-NA is robust enough for application to dose-sparing and adjuvanted vaccines.

## Discussion

The development of a standardized NA quantification method will help the vaccine industry move towards developing vaccines that induce a predictable immune response to NA in addition to HA. The VXI-sNA assay is quantitative, (sub)type-specific, and stability indicating. The assay is available as an off-the-shelf kit that relies on a panel of mAbs that were selected for broad coverage of each seasonal subtype and high specificity, allowing for multiplexed analysis of multivalent influenza vaccines. The selection of multiple broadly reactive mAbs increases the chance that the antibodies will detect new vaccine strains as they emerge since, if antigenic drift does occur, it is unlikely that both epitopes probed by VXI-sNA will change. In the case that antigenic drift renders both of VXI-sNA capture mAbs ineffective for a given subtype, a process is in place for the rapid screening and qualification of a replacement as described previously.^40,41^

It is critical for an NA quantification method to quantify active, immunogenic NA. In our previous study, we demonstrated the correlation of the NA N2 content measured by VXI-sNA with immunogenicity as measured with NAI antibody titers in mice.^31^ In this study, we support this finding by demonstrating the correlation of the VXI-sNA assay with NA activity as measured against a MUNANA-like activity assay. The assay tracked changes in protein stability throughout a forced degradation time course that correlated well with the decrease in activity, suggesting VXI-sNA is quantifying the active from of the protein that is the most relevant for inducing NA immunity.^31^ One limitation to the current study is that the glycosylation status was not determined for any of the samples. Li et al.^42^ previously reported a change in activity of 20-fold for one virus when a glycosylation site was modified and the NAction! taskforce highlighted the role of NA glycosylation in enzymatic activity as deserving further study.^26^ Future studies will explore changes in glycosylation and their effect on quantification using the VXI-sNA assay.

The assay tracked changes in protein stability throughout a forced degradation time course that correlated well with the decrease in activity, further illustrating that VXI-sNA is quantifying the active form of the protein that is the most relevant for inducing NA immunity.^31^

Additional features of the assay include high precision across different users, days, and lots of reagents, as well as an expanded LDR compared to the MUNANA-like activity assay. The low limit of quantification of the assay enables large dilution before analysis, which also dilutes interfering substances such as crude matrix proteins and adjuvants. This feature allows for the use of VXI-sNA for in-process samples, enabling manufacturers to apply the assay to optimize their procedure for the retention of active NA. The compatibility with adjuvants may also enable for the evaluation of the effect of adjuvants on the stability of NA over time.

While the strains from the two B-lineages have antigenically distinct HAs, they encode very similar NAs due to a documented reassortant event.^1,32^ In the presumably rare case where the binding constants for the two B-NA components to the anti-B mAbs used in the VXI-sNA assay are identical, it would be possible to quantify the total B-NA concentration in a quadrivalent sample using the current mAbs. For the B strains investigated here the binding constants are quite different (see Fig. 2); therefore, the VXI-sNA assay in its current form could not be used to quantify the individual B-NA components in a typical quadrivalent vaccine. However, it is important to note that the assay in its current form *is* suitable for trivalent vaccines and for the quantification of monovalent samples generated during all stages of manufacturing prior to final formulation, as demonstrated in this study. To address the individual B-NA components within quadrivalent vaccine, we are seeking mAbs that are able to distinguish between NA from different B strains and are also investigating the potential of competitive binding variations on the current assay.

By utilizing VXI-sNA to assess NA content of historical vaccines (Fig. 1e), we are able to make some observations in regards to the NA content in vaccines over seven flu seasons and 16 production lots. While it is not possible to interpret the significance of differing signal intensities for each vaccine without an available standard, we can compare vaccines from different years that have the same strains—keeping in mind that these vaccines are beyond their official expiration dates. For example, if we focus on the vaccines with B/Brisbane/60/2008, Fluzone 2009–2010, 2010–2011, and 2011–2012, the relative signal intensities vary from 30,720 to 63,958 RFU for antibody BN(i), suggesting the amount of immunogenically active NA varies by as much as 2-fold in these vaccines. Additionally, potentially large variations from lot-to-lot within a given year is evident as shown for the Fluzone 2006–2007 vaccine, of which we tested three distinct lots. The N1(i) capture antibody signal intensity varied from 4011 RFU for Lot U2174EA to 16,566 RFU for Lot U2248AA, a 4.1-fold difference between the two lots. Similar variations were observed for the N2 and B-NA components of the two vaccine lots as well with ~2.0-fold variations in signal intensity from their corresponding capture mAbs from lot-to-lot. On the other hand, the signal intensities of both N2 capture mAbs for the H3N2 A/Uruguay components are relatively consistent for vaccines from 2008–2009 and 2009–2010 across the five production lots tested, suggesting that comparable levels of N2 NA were included in each year (and each lot within each year). The NA from H3N2 viruses has been reported to be more stable than N1 and thus the relatively constant signal observed for N2 NA could be due to the protein’s better stability.^7^ In any case, this analysis appears to support the notion that NA content in commercial vaccines is highly variable,^30^ although such a conclusion cannot be drawn without quantification using a calibration standard. Future studies using VXI-sNA to assess fresh lots of vaccine are needed to address the magnitude of lot-to-lot and year-to-year NA content variation.

If the amount of NA was to be standardized for seasonal influenza vaccines, the VXI-sNA assay could provide manufacturers the ability to optimize their manufacturing process to retain and preserve NA throughout their process. In addition to process optimization for NA retention, manufacturers may also need to supplement with purified NA or recombinant NA. To improve vaccine effectiveness through the standardization of NA, the VaxArray seasonal NA assay can provide information in regards to how much supplemental NA would be required. Taken as a whole, the studies and results described herein demonstrate that VXI-sNA has all of the characteristics needed to empower a significant advancement in influenza vaccines, namely understanding the role of NA in influenza vaccine efficacy and, ultimately, controlling the NA content.

## Methods

### VXI-sNA standard procedure

The VXI-sNA technology is similar to both the VaxArray Influenza Seasonal and Pandemic HA Potency Assays (VXI-sHA and VXI-pHA, respectively).^31,40,43,44^ VXI-sNA reagent kits (Cat # VXI-7300, InDevR Inc.) contain two microarray slides, printed with 16 replicate arrays per slide, Fiducial Detection Label, Protein Blocking Buffer (PBB), and two Wash Buffers. Prior to use, VXI-sNA slides were removed from the refrigerator and equilibrated to room temperature for 30 min in the provided foil pouch. Samples were prepared individually by lysing at room temperature for 30 min in 1× PBS + 0.5% Zwittergent 3-14 (Cat # 693017, EMD Millipore) and 1.5% Triton X-100 (Cat # X-100, Sigma-Aldrich). Each sample was further diluted in Protein Blocking Buffer + 0.5% Zwittergent 3-14 + 1.5% Triton X-100 (PBB/ZT) and 50 µL was applied to individual arrays on the slide following the method described in the VaxArray Influenza Potency Assay Operation Manual (Rev. 001). After a 1-h incubation at room temperature in a humidity chamber (Cat # VX-6200, InDevR Inc.). A label mixture of PBB, Fiducial Detection Label, and antigen detection label was prepared and added to each array following removal of antigen from the slide. Each slide was further incubated for 30 min before subsequent, sequential washing in Wash Buffer 1, Wash Buffer 2, 70% ethanol, and ultrapure water. Slides were dried using a compressed air pump system, imaged using the VaxArray Imaging System (Cat # VX-6000, InDevR Inc.), and raw fluorescent signal intensity of the printed capture antibody spots extracted. Data were automatically processed using either an excel spreadsheet (slightly modified version of the VaxArray Processing Workbook v1.2 described by Kuck et al.^43^) or the VaxArray Analysis Software Package which utilizes the same algorithm described by Kuck et al.^43^ For each capture antibody, calibration curves were plotted using antigen concentration and median signal intensity of the nine antibody replicate spots on each individual array, and, when appropriate, the NA concentration of samples was automatically calculated against the standard curve.

### Antigen detection labels used in this study

The NA A&B pAb Label (InDevR, Cat # 7616), a “universal” NA polyclonal label, was used for all egg-derived antigens and alternative mAb labels (InDevR, Cat # VXI-7612 and VXI-7615) were used for the detection of non-egg-derived antigens. All antibodies are commercially available and were used at a final 1× concentration as recommended by the manufacturer in all VaxArray experiments. The need for different labels is possibly due to differences in glycosylation patterns. The “universal” polyclonal antibody was raised against egg-derived antigens and has consistently under performed in labeling cell-derived antigens. The alternative mAb label antibodies were previously developed for broad detection regardless of antigen production method.

### Evaluation of coverage over time and antigenic drift

A panel of Fluzone TIVs (provided by BEI Resources, Lot #s reported in Fig. 1e) were analyzed by VXI-sNA following the standard procedure. Each sample was individually lysed in PBS + 0.5% Zwittergent and 1.5% Triton, further diluted in PBB + 1.5% Triton and 0.5% Zwittergent (PBB/ZT) to 3 µg/mL HA from each subtype/type. Samples were labeled with the NA A&B pAb Label (InDevR, Cat # 7616) and imaged using the VaxArray Imaging System and Software.

### NA activity assay

The NA Activity Assay was performed according to the product technical sheet for the NA Activity Assay Kit from Sigma-Aldrich (Cat # MAK121). Briefly, a standard curve of standardized enzyme, provided in the kit, was serially diluted in water to 80, 48, 24, and 0 µM. Twenty microliters of each sample was added to a clear flat-bottom, black-walled optical grade 96-well plate (Cat # 265301, ThermoFisher) in replicates of 6 and standards were added in triplicate. A reaction mix (with substrate) as well as a blank reaction mix (no substrate) was prepared. Eighty microliters of reaction mix was added to half of the sample replicates and all of the standard replicates. To the remaining half of the sample replicates, 80 µL of the blank reaction mix was added. The plate was sealed with optical grade film (Cat # 4313663, ThermoFisher) and incubated in an Optima FLUOstar 96-well plate reader with a holding temperature of 37 °C with intermittent shaking. Every 120 s, wells were excited at 530 nm and the associated fluorescent emission at 570 nm was measured for a 90-min period. The fluorescent intensity of the standard wells at *t* = 50 min was plotted against the corresponding standard concentrations. The standard curve was evaluated for linearity, the slope of the linear regression was calculated, and the activity of each sample was determined as described in the manufacturer’s instructions.

### LDR and correlation with activity assay

NA containing samples were individually diluted in PBS to 7 µg/mL of NA in PBS and further serially diluted in PBS to generate a 13-point dilution series. Each dilution was split, with half being processed by the VaxArray standard procedure with lysis in PBS + 0.5% Zwittergent and 1.5% Triton along with three antigen-blank arrays, and the other half being analyzed by the NA activity assay under the standard procedure described above. Each dilution and sample was analyzed at the same final concentration by both VaxArray and the activity assay. Fluorescent signal intensity was measured for VXI-sNA using the VaxArray Imaging System and Software. Sample activity was calculated following the manufacturer’s instructions. For VXI-sNA, the lower limit of quantification was calculated from the signal intensity of the antibody in an antigen-blank well plus five times the standard deviation of the antibody signal in that array. This signal was converted to an antigen concentration using the lowest quantification range of the antibody. A lower QL was calculated for each antigen-blank array and averaged. The upper QL of VXI-sNA was defined as the highest concentration of the highest 4-point quantification range that has an *R*^2^ value >0.95. For the activity assay, the manufacturer has a stated lower limit of quantification of 0.01 U/L. The linear regression calculated for the activity response curve was used to convert this activity QL to an NA concentration for each sample tested. The upper QL was defined as the highest concentration tested whose activity assay response fell within the standard curve of the assay. The LDR for each assay was defined as the upper QL divided by the lower QL.

### Assay precision determination

A trivalent mixture of H1N1, H3N2, and BY reference antigens (CBER Lots 76, 84, and 80, respectively) was diluted in PBS/ZT. A serial dilution of antigen was prepared in PBB/ZT to generate a standard curve starting at 0.4, 0.1, and 0.3 µg/mL of N1, N2, and B-NA antigen, respectively. The lysed sample was also diluted in PBB/ZT separately to three separate dilutions within the low (0.08 µg/mL N1, 0.02 µg/mL N2, 0.06 µg/mL B-NA), middle (0.16 µg/mL N1, 0.04 µg/mL N2, 0.12 µg/mL B-NA), and upper (0.32 µg/mL N1, 0.08 µg/mL N2, 0.24 µg/mL B-NA) regions of the curves. Samples were analyzed in eight replicates by VXI-sNA using the standard procedure and labeled with the NA A&B pAb Label (InDevR, Cat # 7616). The experiment was repeated on three separate days, by three separate operators, using three separate lots of reagents (slide lots, label lots, PBB lots, and wash buffer lots). Samples were quantified against the appropriate standard curve and the NA concentration of each sample and replicate was determined.

### Thermal degradation and stability indication

A B/Phuket/3073/2013 reference antigen (CBER Lot # 80) was diluted to 30 µg/mL HA in PBS. Eight separate, identical aliquots were added to amber glass vials, sealed with crimp tops, and incubated in a 45 °C water bath for up to 10 h. For VXI-sNA, samples were lysed in PBS + 0.5% Zwittergent and 1.5% Triton (PBS/ZT) before further dilution in PBB/ZT to 1.4 µg/mL and analyzed in triplicate by VXI-sNA using the standard procedure. A standard curve, generated using the non-degraded (4 °C, T0) aliquot, was used to calibrate the assay. All samples were labeled with the NA A&B pAb Label (InDevR, Cat # 7616). Samples were also diluted in ultrapure water to 1.4 µg/mL of HA and analyzed using the NA activity assay under the standard procedure described above.

### Quantification of NA in crude in-process samples

A trivalent mixture of H1N1, H3N2, and BY reference antigens (CBER Lots 76, 84, and 80, respectively) was spiked into a 40% sucrose solution (Cat # S9378, Sigma-Aldrich), mock-infected allantoic fluid from 10-day-old embryonated chicken eggs (Cat # BV027, Virapur), and exhausted Dulbecco’s modified Eagle’s medium (DMEM) + 10% fetal bovine serum (FBS) tissue culture media taken from non-infected MDCK cells (provided by collaborator) to mock 2.5 µg/mL of HA (0.44 µg/mL of N1, 0.24 µg/mL of N2, and 0.40 µg/mL of B-NA based upon the IDMS-determined NA concentration of each of the stock antigens) in each matrix. Spiked samples were lysed in PBS/ZT and further diluted in PBB/ZT to a final expected concentration of 0.4 µg/mL of HA from each antigen (0.07 µg/mL of N1, 0.04 µg/mL of N2, and 0.06 µg/mL of B-NA). A standard curve of non-crude matrix spike antigen was prepared by lysing the same original trivalent mixture of antigens in PBS/ZT, diluting to 1 µg/mL HA (0.18 µg/mL of N1, 0.10 µg/mL of N2, and 0.16 µg/mL of B-NA) in PBB/ZT, and then further serially diluting in PBB/ZT. These calibration standards were analyzed alongside the crude matrix-spiked antigens, analyzed in quadruplicate, by VXI-sNA. The NA concentration of each replicate of each spiked sample was compared to the expected sample concentration based on the known NA concentration of the stock antigen and the performed dilutions.

### Quantification of NA in low-dose adjuvanted vaccines

A trivalent mixture of H1N1, H3N2, and BY reference antigens (CBER Lots 76, 84, and 80, respectively) was added to aluminum hydroxide (Cat # 77161, ThermoFisher), MF59 (Cat # Vac-adx-10, Invivogen), and PBS at a final concentration of 5.0 µg/mL of HA each (0.87 µg/mL of N1, 0.48 µg/mL of N2, and 0.80 µg/mL of B-NA based upon the IDMS-determined NA concentration of each of the stock antigens), resulting in two “mock” adjuvanted vaccines with adjuvant concentrations of 0.17 mg/mL of elemental aluminum or 1.95 mg/mL of squalene and a negative PBS control. Each solution was incubated at room temperature for 30 min to mimic the bed-side mixing of adjuvanted vaccines in practice. After 30 min, the adjuvanted and PBS control samples were lysed in PBS/ZT for 30 min and further diluted in PBB/ZT to 0.4 µg/mL of HA from each antigen (0.07 µg/mL of N1, 0.04 µg/mL of N2, and 0.06 µg/mL of B-NA). A standard curve of non-adjuvanted antigen was prepared by lysing the PBS control sample in PBS/ZT, diluting to 1 µg/mL HA (0.18 µg/mL of N1, 0.10 µg/mL of N2, and 0.16 µg/mL of B-NA) in PBB/ZT with a further serial dilution in PBB/ZT and was analyzed alongside the adjuvanted samples, analyzed in quadruplicate, by VXI-sNA. The NA concentration of each replicate of each adjuvanted sample was compared to the expected sample concentration based on the known NA concentration of the stock antigen and the performed dilutions.

### Reporting summary

Further information on experimental design is available in the Nature Research Reporting Summary linked to this article.

### Code availability

The mathematically algorithm used to analyze the VXI-sNA assay can be accessed in a previously published study.^43^

## Supplementary information

Supplementary Information

Reporting Summary

## Data Availability

All relevant data from this study are available from the authors.

## Supplementary information

**Supplementary information** accompanies the paper on the *npj Vaccines* website (10.1038/s41541-019-0099-3).
