# Supplementary material for: A neuraminidase potency assay for quantitative assessment of neuraminidase in influenza vaccines
Source: NPJ Vaccines. 2019 Jan 22;4:3. doi: 10.1038/s41541-019-0099-3 (PMC6342948; doi:10.1038/s41541-019-0099-3)
Supplement: Supplementary file 1 — Supplementary Information [file 41541_2019_99_MOESM1_ESM.pdf]

SUPPLEMENTARY INFORMATION

Antibody Screening for Down-Selection of VXI-sNA Capture Antibodies. A panel of 21 monoclonal antibodies (11 anti-N1, 2 anti-N2, and 8 anti-BNA) was assembled from materials provided by our collaborators at Mt. Sinai School of Medicine as well as commercial sources. Each antibody was “printed” in replicates of three on a VaxArray screening array. The anti-N1 and N2 antibodies were printed on one lot of slides and the anti-BNA antibodies were printed on another. Each slide lot had its corresponding array printed in 16 wells on a modified glass substrate using the same parameters utilized for the other VaxArray technologies. To evaluate mAbs for specificity and coverage, a test panel of 12 influenza A antigens, 3 influenza B antigens, and 3 seasonal trivalent influenza vaccines (TIVs) were diluted to 3 µg/mL HA (NA concentrations were unknown) and added individually to the N1/N2 array. A panel of 2 influenza B antigens, 4 influenza A antigens, and 3 TIVS were diluted to 3 µg/mL HA and added to the BNA array. Cross-reactivity was also evaluated by adding the B antigens to the “A microarray” and vice versa. Each well was labeled with a polyclonal antibody label (InDevR, Cat # VXI-7601). The resulting fluorescent signal from each antibody “spot” within the array was measured by the VaxArray Imaging System (InDevR, Cat # VX-6000) and the raw intensity values were averaged for each capture mAb. The resulting average relative fluorescent units were compared for each antigen on each capture mAb and summarized in Supplementary Figure 1ab. As shown in the Supplementary Figure 1a, anti-N1 mAbs 2, 6, and 8 demonstrated some levels of cross-reactivity, interacting with H3N2 antigens. None of the anti-N1 and anti-N2 mAbs reacted with B antigens. Of the anti-N1 mAbs, 4 and 11 demonstrated 100% coverage (detecting all strains tested within the N1 subtype) and 100% specificity (not detecting any non-N1 samples). Anti-N2 mAbs, 12 and 13, and anti-BNA, mAbs 20 and 21, also demonstrated 100% coverage and specificity.

The antibodies downselected for coverage and specificity were assessed for stability indication capabilities. References antigens representing H1N1, H3N2, and BV subtypes were analyzed in triplicate (n=3) by VXI-sNA before (T0) and after 20 hours incubated at 45°C (T20). %T0 values for each sample (average signal at T20 divided by average signal at T0) were calculated. As demonstrated in Supplementary Figure 2, all antibodies reported reductions in signal intensities of 37 – 97% upon heat degradation, suggesting the downselected mAbs only bind properly folded NA and not degraded protein.

a

Strain Information		Relative Signal Intensities: Anti-N1 mAbs											Relative Signal Intensities: Anti-N2 mAbs		Antigen Information		
Subtype	Name	mAb 1	mAb 2	mAb 3	mAb 4	mAb 5	mAb 6	mAb 7	mAb 8	mAb 9	mAb 10	mAb 11	mAb 12	mAb 13	Type	Source	Lot
H1N1	A/New Caledonia/20/1999														egg, refAg	CBER	56
H1N1	A/Brisbane/59/2007														rNA	BEI	NR-43785
H1N1	A/California/04/2009														rNA	BEI	NR-19234
H1N1	A/California/07/2009														egg, refAg	CBER	76
H3N2	A/Wisconsin/67/2005														rNA	ATCC	NR-19237
H3N2	A/Brisbane/10/2007														egg, refAg	CBER	2007/79B
H3N2	A/Victoria/210/2009														egg, refAg	CBER	70
H3N2	A/Victoria/361/2011														egg, refAg	CBER	73
H3N2	A/Texas/50/2012														egg, refAg	CBER	75
H3N2	A/Switzerland/9715293/2013														egg, refAg	CBER	82
H3N2	A/Hong Kong/4801/2014														egg, refAg	CBER	84
BY	B/Massachusetts/02/2012														egg, refAg	CBER	74
BY	B/Phuket/3073/2013														egg, refAg	CBER	80
BV	B/Brisbane/60/2008														egg, refAg	CBER	77
TIV	Fluzone 2010-2011 TIV														vaccine	BEI	NR-X
TIV	Agriflu 2010-2011 TIV														vaccine	BEI	NR-X
TIV	afluria 2010-2011 TIV														vaccine	BEI	NR-X

b

Strain Information		Relative Signal Intensities: Anti-B mAbs								Antigen Information		
Subtype	Name	mAb 14	mAb 15	mAb 16	mAb 17	mAb 18	mAb 19	mAb 20	mAb 21	Type	Source	Lot
BV	B/Brisbane/60/2008									egg, refAg	CBER	77
BY	B/Massachusetts/02/2012									egg, refAg	CBER	74
H1N1	A/California/04/2009									rNA	BEI	NR-19234
H1N1	A/California/07/2009									egg, refAg	CBER	76
H3N2	A/Wisconsin/67/2005									rNA	ATCC	NR-19237
H3N2	A/Hong Kong/4801/2014									egg, refAg	CBER	84
TIV	Fluzone 2010-2011 Trivalent									vaccine	BEI	NR-X
TIV	afluria 2008-2009 Trivalent									vaccine	BEI	NR-X
TIV	FLUARIX 2008-2009 Trivalent									vaccine	BEI	NR-X

Supplementary Figure 1. Antibody Screening for Down-Selection of VXI-sNA Capture Antibodies. The listed panel of antigens in **(a)** were tested on the VXI-sNA Screening array A and the panel of antigens in **(b)** were tested on the VXI-sNA Screening array B. White/empty boxes indicate signal intensity below the average array background plus 3 standard deviations of the background (3878 RFU). The blue color intensity is presented as a scale from 3878 RFU to fluorescence saturation. Downselected antibodies are highlighted yellow.

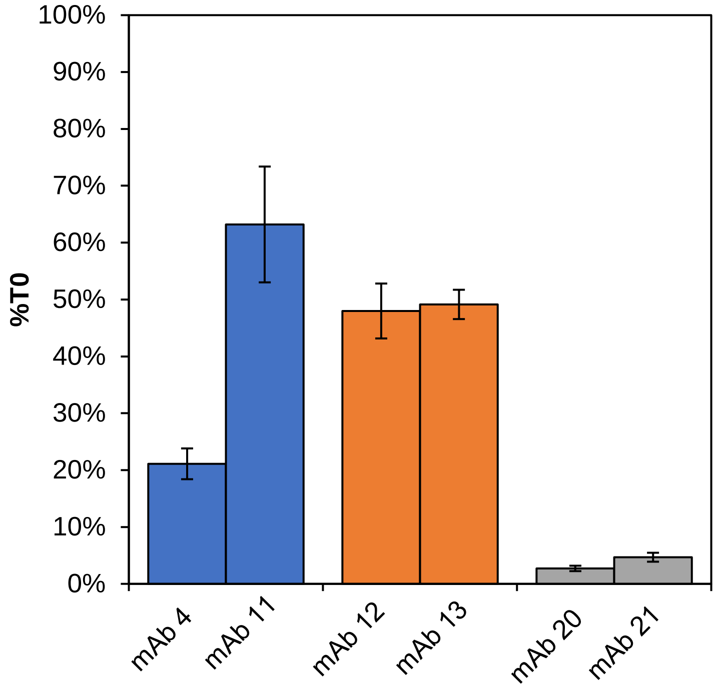

Supplementary Figure 2. VXI-sNA Capture mAbs are Stability Indicating. Reference antigens for H1N1 A/California, H3N2 A/Hong Kong, and B/V B/Brisbane (CBER, Lots #76, 84, 77, respectively) were degraded for 0 and 20 hours and analyzed by VXI-sNA in triplicate. The measured signal intensity of the degraded antigen samples (T20) was divided by the measured signal intensity of the non-degraded antigen samples (T0) and plotted (%T0) for the N1 capture mAbs (blue bars), N2 capture mAbs (orange bars), and BN capture mAbs (grey bars) for the appropriate antigen. Error bars represent the propagated error (standard deviation) of the triplicate measurements of the degraded and non-degraded antigen samples.

VXI-sNA detects NA and not HA. Because NA is often at much lower concentrations than HA in vaccine formulations, we wanted to demonstrate that VXI-sNA was only quantifying NA and not affected by the much higher concentrations of HA in vaccines. To this end, recombinant H1, H3, B-Yamagata-like and B-Victoria-like HA proteins and a quadrivalent mixture of the four proteins were analyzed on VXI-sNA and the VaxArray Influenza Seasonal HA assay (Supplementary Figure 3ab). The VaxArray HA assay was able to detect the recombinant HA proteins with robust signal. In contrast, the same recombinant HA formulations were not detected by the VaxArray NA assay, as expected. The signal intensities for each NA capture mAb was below the limit of quantification for the assay, suggesting that VXI-sNA is specific for NA and not affected by the presence of high concentrations of HA.

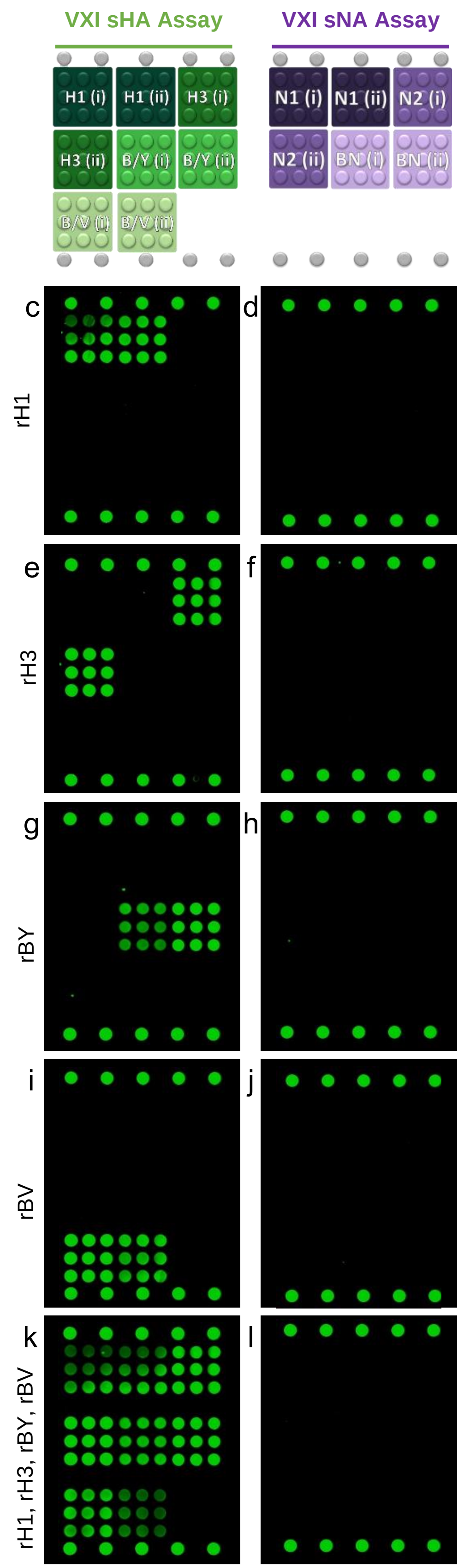

Supplementary Figure 3. VXI-sNA protein specificity. A panel of recombinant HA proteins were analyzed by both the VXI-sNA and VXI-sHA (seasonal HA) assays. Array layouts for VXI-sHA **(a)** and VXI-sNA **(b)** are shown. Array images for VXI-sHA and VXI-sNA and the measured signal of each antibody, respectively, are shown for recombinant H1 **(c, d)**, recombinant H3 HA **(e,f)**, recombinant BY HA **(g, h)**, recombinant BV HA **(i,j)**, and a quadrivalent mixture of recombinant H1, H3, BV, and B/Y HA **(k,l).**

SUPPLEMENTARY METHODS

Antibody Screening for Down-Selection of VXI-sNA Capture Antibodies. Monoclonal antibodies were printed in triplicate in 16 replicate arrays on a microarray slide (Fig. 2a). Antigens were lysed in PBS + 1% Zwittergent 3-14 for 30 minutes and further diluted to 3 µg/mL of HA in PBB + 1% Zwittergent 3-14, before analysis using the standard VaxArray procedure described above. Each antigen was labeled with a "universal" neuraminidase-reactive polyclonal label antibody (NA A&B pAb Label, Cat # VXI-7616, InDevR). Coverage for a given antibody is defined as the number of subtype/type specific detection events (greater than 3x background signal intensity) divided by the number of subtype/type specific antigens evaluated against the antibody. Specificity for a given antibody is defined as the number of subtype/type specific detection events divided by the total number of detection events of the antibody.

Determination of Antibody Stability Indication Capabilities

Reference antigens (A/California/07/2009 (CBER Lot #76), A/ Hong Kong/4801/2014 (CBER Lot #84), B/Brisbane/60/2008 (CBER Lot #77)) were diluted to 30 µg/mL of HA in PBS, aliquoted into separate glass vials, sealed, and one vial of each antigen was incubated in a 45°C water bath for 20 hours. A non-degraded, control aliquot of each antigen was stored at 4°C for the duration of the experiment. T0 (control) and T20 (degraded at 45°C for 20 hours) samples for each antigen were evaluated with standard VaxArray procedure by VXI-sNA in triplicate at a final non-degraded concentration of 1.5 µg/mL HA, labeled with the universal NA polyclonal antibody (InDevR, Cat # VXI-7616). Stability indication is defined as the ability to detect changes in protein stability/conformation, indicated by a drop in antibody signal in response to degradation or deterioration of antigen.

Influenza Protein Specificity Determination. Samples containing recombinant H1 (CBER, Lot # H1-Ag-1303), H3 (ImmuneTech, cat# IT-003-00423ΔTM), B/Y (ImmuneTech, cat# IT-003-B7ΔTM), and B/V HA (BEI, cat# NR-19239) were lysed in PBS + 1% Zwittergent monovalently, and as a quadrivalent mixture and diluted to 10 µg/mL in PBB/Z. Samples were analyzed by both the VaxArray Influenza Seasonal Hemagglutinin Potency Assay (VXI-sHA, Cat # 7101, InDevR) and VXI-sNA using the standard procedure. Recombinant H1 protein was labeled with a monoclonal broad-HA specific antibody label (Cat # VXI-7604, InDevR), recombinant H3 protein with a monoclonal H3-specific antibody label (Cat # VXI-7610, InDevR), recombinant B/Y HA protein with a monoclonal B/Y HA-specific antibody label (Cat # VXI-7607, InDevR), and recombinant

B/V protein with a monoclonal B/V HA-specific antibody label (Cat # VXI-7606, InDevR). The multivalent antigen arrays were labeled with a mixture of these four labels.
